# Unraveling and Sliding of Polypeptide Strands Underlies the Exceptional Toughness of the Triple-Helix Collagen Molecule

**DOI:** 10.1021/acsnano.5c15873

**Published:** 2026-01-09

**Authors:** Andreas Rohatschek, Bruno Zappone, Patrick Steinbauer, Manuel Rufin, Daniela A. Barrágan Rivera, Maria P. De Santo, Orestis G. Andriotis, Stefan Baudis, Philipp J. Thurner

**Affiliations:** 1 Institute of Lightweight Design and Structural Biomechanics, TU Wien,Gumpendorfer Straße 7/Objekt 8, Vienna 1060, Austria; 2 Consiglio Nazionale delle Ricerche - Istituto di Nanotecnologia (CNR-Nanotec), Via P. Bucci 33/C, Rende (CS) 87036, Italy; 3 Institute of Applied Synthetic Chemistry, Division of Macromolecular Chemistry, 27259 TU Wien, Vienna 1060, Austria; 4 Christian Doppler Laboratory for Advanced Polymers for Biomaterials and 3D Printing, 27259 TU Wien, Vienna 1060, Austria; 5 18950 Università della Calabria - Dipartimento di Fisica, Via P. Bucci 31/C, Rende (CS) 87036, Italy; 6 Biointerface Doctoral School, 27259 TU Wien, Vienna 1060, Austria; 7 Austrian Cluster for Tissue Regeneration, Vienna 1200, Austria

**Keywords:** collagen, AFM, SFA, force spectroscopy, mechanical properties

## Abstract

Fibril-forming tropocollagens (TCs) play an essential role in tissue biomechanics. They are ubiquitous in mammals and other animal tissues, where they provide passive mechanical functions. While molecular dynamics simulations have targeted the mechanics of individual TCs, experimental data on their tensile mechanical properties remain scarce. As a consequence, the link between the unique triple-helix structure of the collagen molecule and macro-mechanical properties of collagenous tissues is not well understood. To close this gap, we have investigated isolated TCs grafted on the tip of atomic force microscopy (AFM) probes as well as adsorbed TC films using a surface force apparatus (SFA). AFM force spectroscopy showed that an individual TC can be stretched without failing to a contour length of up to 900 nm—nearly three times its native length—over thousands of stretching cycles. The molecule was retracted from a strongly adhering mica surface by pulling on one of the α-chains, forcing the triple-helix to unravel. During this process, the α-chains slipped progressively, irreversibly, and almost entirely past each other before being caught by strong physical interactions between overlapping chain ends. SFA measurements showed that strong electrostatic interactions bind TC to mica and prevent TC aggregation, supporting the AFM results. These findings indicate that a controlled slippage mechanism underpins the exceptional toughness of TCs, collagen fibrils, and collagen-rich tissues such as tendons and skin.

## Graphical abstract

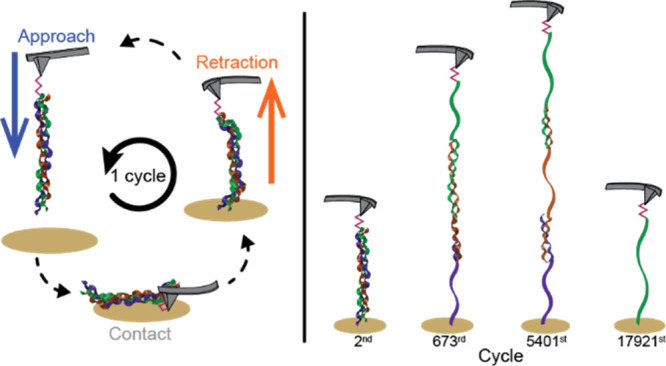

Collagen-rich tissues are simultaneously soft and tough, i.e., they undergo large mechanical deformations and damage under tension but maintain structural cohesion without completely failing.^1^ For example, energy-storing tendons can be extended in tension by up to 20% prior to failure,^2^ and collagen fibrils, the supra-molecular assemblies built from collagen molecules, show tensile failure strains larger than 35%.^3^

At the smallest length scale, the structure and mechanical properties of collagen tissue originate from the unique triple helix structure of fibril-forming collagen molecules, also known as tropocollagens (TCs), which are the most abundant and widespread collagen subfamily in the human body.^4^ The triple helix is formed from three α-chains, conformational P_II_-spirals,^5^ comprising about 1000 amino acids (aa) each. The chains contain the characteristic triplet amino acid repeat sequence Glycine-X-Y, where X and Y are often Proline or Hydroxyproline, stabilizing the triple-helix via numerous hydrogen bonds. In type-III collagen, the triple helix comprises three identical α-chains and occupies the central portion of the molecule, flanked by short nonhelical regions.^6^ The molecule has a diameter of about 1.5 nm and a length of about 300 nm.^7^

Although TC deformations caused by tension and shear loading are particularly relevant to the toughness and ultimate tensile strain in collagen-rich tissues, they have not been fully characterized experimentally. Yet, establishing a clear connection between the mechanical properties and behavior of TCs and collagenous tissues is essential to biology, medicine, and biomaterial development. For example, mutations related to collagen-coding genes cause severe diseases such as Osteogenesis Imperfecta^8^ or Ehlers-Danlos syndrome.^9^ Notably, experimental studies at the molecular level^10–12^ and numerical simulations using molecular dynamics (MD) indicate that shear loading causes TC denaturation.^13–16^

We hypothesize that mechanical degeneration of TC is an important process that provides toughness to collagen-rich tissues. Our study addresses this hypothesis directly by measuring the molecular elongation and tensile force of a recombinant human type-III TC. The molecule was attached near the *N*-terminus to a NHS-PEG_27_-MI (maleimidopropionyl-polyethylene glycol-hydroxysuccinimide ester) linker grafted to a silicon-oxide AFM probe tip, using established bioconjugation protocols (Figure 1a).^17,18^ Specifically, a thiol-based bioconjugation method was used to target one of the two cysteine residues close to the N-terminus of the α-chains (amino acid numbers 1043 and 1044). Therefore, this site-specific bioconjugation allows attaching the linker to one of the six cysteine residues of type-III TC.

**Figure 1:**
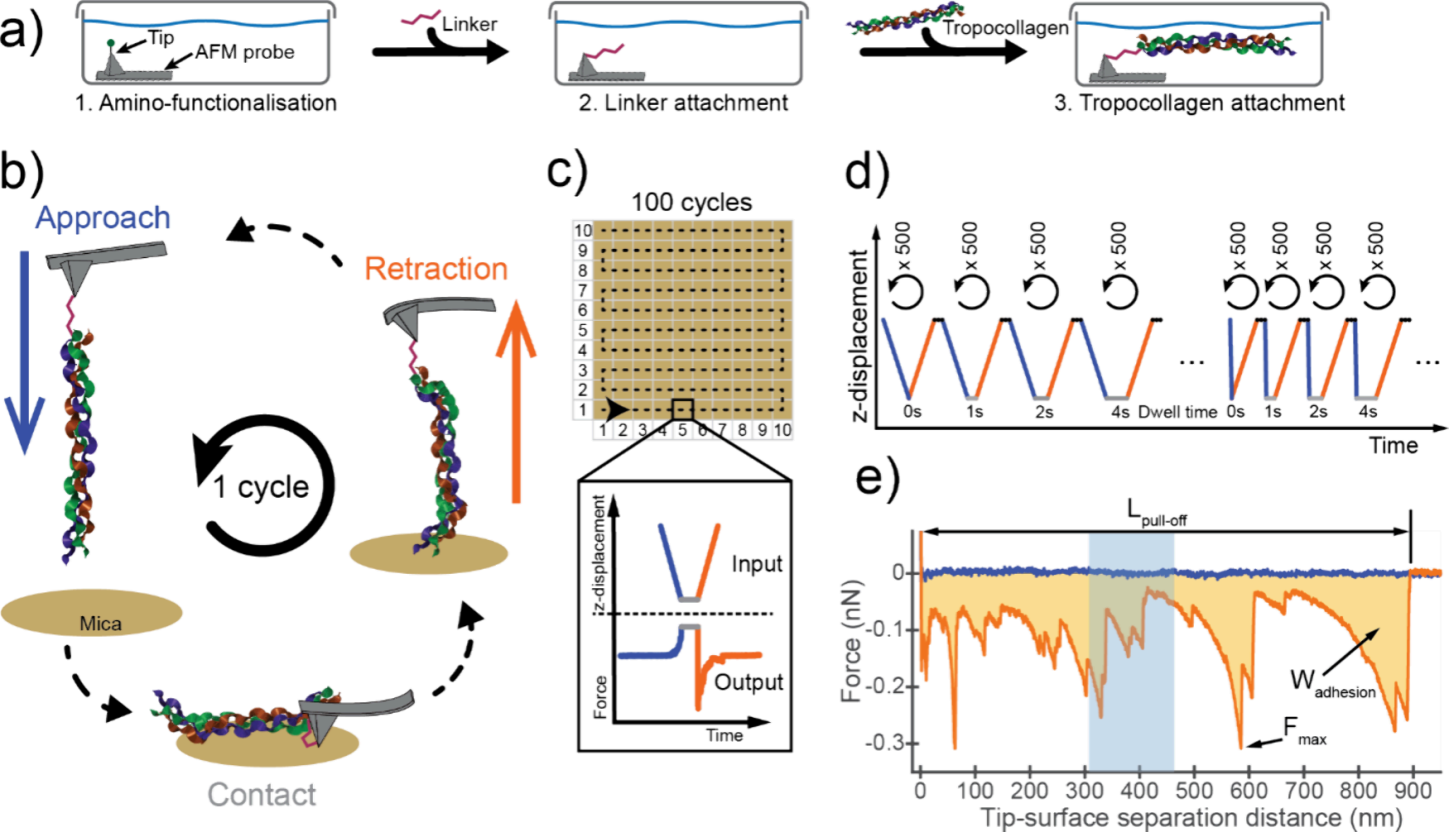
Design of the AFM experiments. (a) Three-step bioconjugation protocol used to covalently attach TC to the AFM probe. (b) Illustration of an AFM measurement cycle showing approach (blue), dwell time in contact (gray), and retraction (orange) between a TC-functionalized AFM probe and a muscovite mica surface measured in acetic acid solution at pH 3.5. (c) A set of 100 cycles was obtained with constant experimental parameters on a 10 × 10-point square grid with 10 × 10 μm^2^ surface area. The inset shows the vertical displacement imposed on the AFM cantilever and the measured force. Approach, retraction, and dwell time in contact are shown in blue, orange, and gray, respectively. (d) Example of variation in approach/retraction speed (curve slope) and dwell time. These parameters were held fixed for 500 cycles. Different line slopes correspond to the speeds of 0.1, 0.25, 0.5, 1.0, 1.5, or 2.0 μm/s. The length of the gray line corresponds to the dwell time (0 s, 1 s, 2 s, 4 s). (e) Example of an AFM force–distance curve obtained from an AFM measurement cycle. The measured force (*y*-axis) is presented as a function of the tip–surface separation distance (*x*-axis). The determined parameters are the pull-off length (*L_pull‑off_*), maximum adhesion force (*F_max_*) and work of adhesion (*W_adhesion_*), shaded in yellow. Blue and orange lines represent the approach and retraction part of a cycle, respectively. The blue vertical band from 308 to 463 nm shows the expected length of the TC-linker complex.

Several thousand AFM force–distance cycles were obtained in acetic acid solution by approaching, keeping in contact, and retracting the attached TC relative to a freshly cleaved mica surface (Figure 1b). The measurements were averaged over 100 different surface positions to minimize the effect of surface inhomogeneities (Figure 1c). The effect of dwell times as well as the approach and retraction speed was investigated. In total, 47 combinations of approach-retraction speeds and dwell times were used (Figure 1d).

TC adhered to mica during tip–surface contact and was stretched upon retraction, resulting in a random sequence of (negative) adhesive force peaks smaller than 1.5 nN, ending with a sudden drop to zero force upon complete pull-off and detachment from the surface (Figure 1e). Multiple adhesive peaks were obtained as long as TC was attached to the AFM tip over thousands of cycles (Figure 3a), a behavior not observed prior to TC attachment (Figure S2) or with different solvents and surfaces (Figure S3). These adhesive force peaks are typical for linear molecules (*e.g.,* proteins, DNA) being stretched and pulled from an adhering surface.^19^ A peak corresponds to stretching the molecule to the rupture point of one or multiple physical bonds within the molecule or between the molecule and the surface. In our experiments, adhesive force peaks were observed over many cycles because the TC repeatedly adhered to the mica surface during tip–sample contact.

## Results and Discussion

### SFA Measurements of TC-TC and TC-Mica Interaction

To elucidate the nature of the TC-mica interactions, the surface forces apparatus (SFA)^20,21^ was used in combination with AFM imaging (see ″SFA force–distance measurements″ and ″AFM measurements of adsorbed TC layers″ in Supporting Information - Supporting Information). AFM images revealed abundant TC adsorption on mica, producing layers with compact and uniform surface morphology without holes, patches, or fiber-like features (Figure S5).

SFA experiments were performed in acetic acid by either pressing an adsorbed TC layer against an uncoated mica surface or another TC layer (see ″SFA force–distance measurements″ in SI). These experiments revealed that the normal force *F* between two TC layers was purely repulsive and followed an exponential decay for mica–mica separation distances *D* in the range (10–70) nm, with an initial amplitude *A* = (5–10) mN/m and a decay length λ = (12–20) nm ([Fig fig2]a). This length is comparable to the Debye length of the electrolyte solution used in our experiments (hydrochloric acid or acetic acid, see ″SFA force–distance measurements″ in SI). Therefore, the repulsive force *F* measured at large distances between equally charged TC was due to electrostatic repulsion. These electrostatic interactions were caused by the numerous positively charged amino acids ot type-III TC. Indeed, these charges account for its high isoelectric point (IEP = 9.36).^22^ In contrast, mica is negatively charged at pH > 3.^23,24^ In a 1:1 electrolyte at room temperature, the repulsive force between two equally charged surfaces is approximately equal to *F/R* = (*Z*/*λ*)­e^–*D*/*λ*^ where *R* is the radius of curvature of the surfaces, *Z* = *C*tanh^2^(*ψ_0_*/103 mV) depends on the surface potential *ψ_0_*, and *C* = 9.22 × 10^–11^ N.^20,21^ The approximation is valid for *ψ_0_* < 100 mV and entails *Z* ≤ *C*. In our experiments, *Z* = *A* *λ* reached values as high as 10^–10^ N and was larger than *C*. This result shows the surface potential of an adsorbed TC layer was particularly high, i.e., *ψ_0_* ≥ 100 mV.

**Figure 2:**
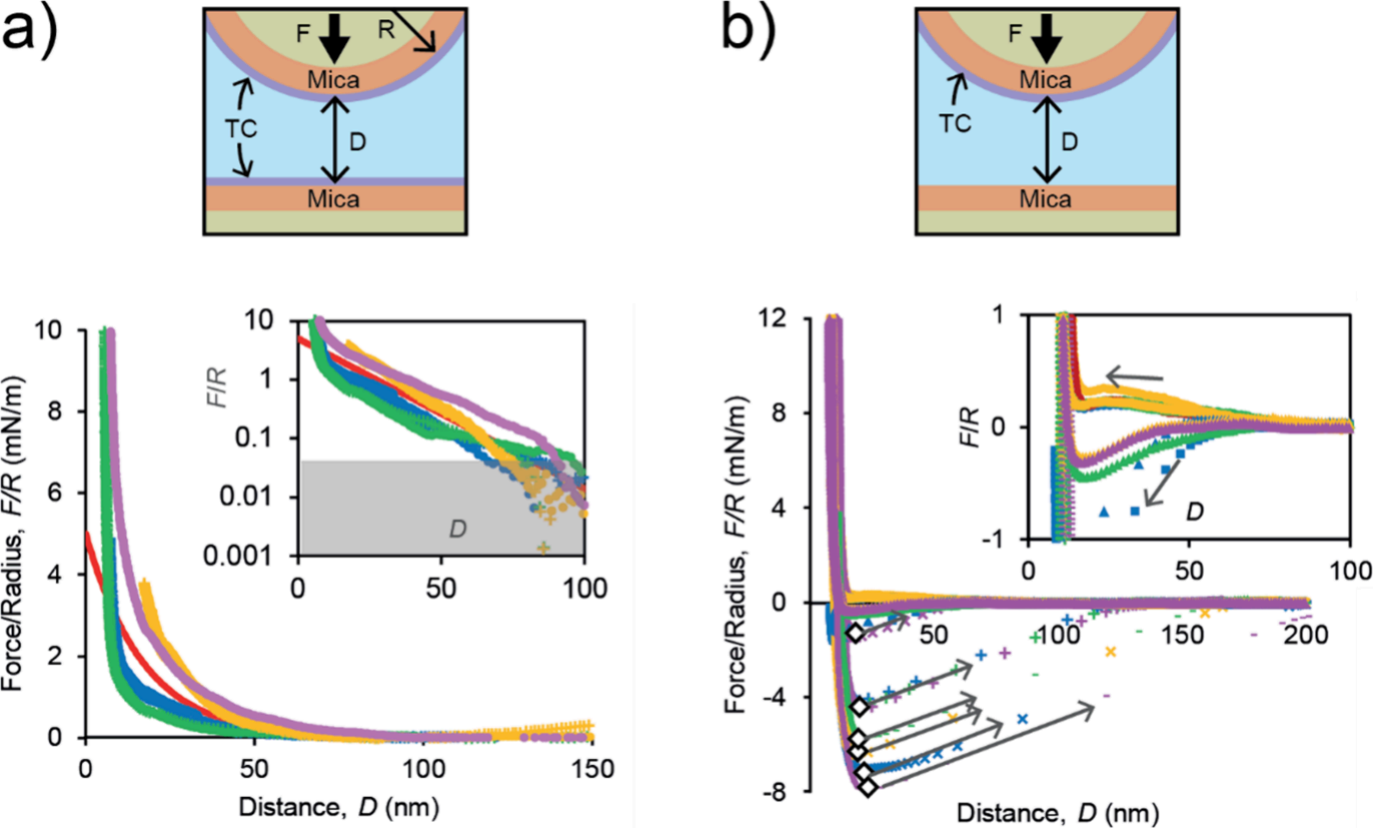
Normal forces *F* generated by collagen type-III layers adsorbed on crossed cylindrical mica surfaces with radius *R* = 2 cm. *D* is the mica–mica separation distance. (a) Interaction between two TC layers. Each color corresponds to one force measurement obtained by approaching (filled symbols) and retracting the surfaces (crosses). For all measurements, the approach and retraction speed in the noncontact region was about 4 nm/s. The inset shows the semilogarithmic plot of *F/R* vs *D*, where the shaded area is below the SFA force accuracy limit. The red solid line represents the electrostatic repulsion *F/R* = *Ae* ^−*D*/*λ*^ with Debye length *λ* and amplitude *A*. (b) Interaction of a TC layer with uncoated mica surfaces. The arrows indicate surface approach or retraction, and white diamonds show the points (*D*_*a*_, *F*_a_) where mechanical instability (jump out) developed during retraction. The inset shows an enlarged view of the weak forces measured during approach. The adhesion energy is *W_adhesion_* = (2/3π)*F*_*a*_/*R*.

Conversely, the force between a TC layer and an uncoated mica surface was strongly adhesive (Figure 2b). Adhesion introduced an instability in the TC layer response to surface retraction.^21^ Namely, the surfaces rapidly jumped from the point of maximum adhesive force at a small distance toward a zero-force region at large distances. During surface retraction, a single adhesive peak was observed with a normalized adhesion force *F/R* = (4–8) mN/m, corresponding to an adhesion energy of (0.8–1.7) mJ/m^2^. These values are quite large and comparable to the strong adhesion generated by mussel adhesive proteins.^25^ Therefore, attractive electrostatic interactions favor TC adsorption on mica, possibly in cooperation with other types of interaction (*e.g.*, hydrogen bonds, hydrophobic). The repulsion between TC layers also shows that TCs do not attract each other and do not aggregate in acetic acid solution.

### TC Denaturation over Thousands of Stretching Cycles

All AFM measurements were obtained in the (28–31)°C temperature range. Force–distance measurements were characterized by the snap-in length (*L_snap‑in_*) and maximum attractive force (*F_max,attractive_*) measured during approach (Figure S4) as well as the pull-off length (*L_pull‑off_*), work of adhesion (*W_adhesion_*), and maximum adhesion force (*F_max_*) measured during retraction (Figure 1e). Although these parameters varied significantly from one cycle to the next, averaging over multiple cycles revealed a consistent evolution of the parameters as the number of cycles increased (Figure 4a-b). Importantly, the SFA experiments described above showed that the increase in pull-off length observed in AFM force–distance curves (Figure 3a, Figure 4a) was not due to the formation of TC aggregates.

**Figure 3:**
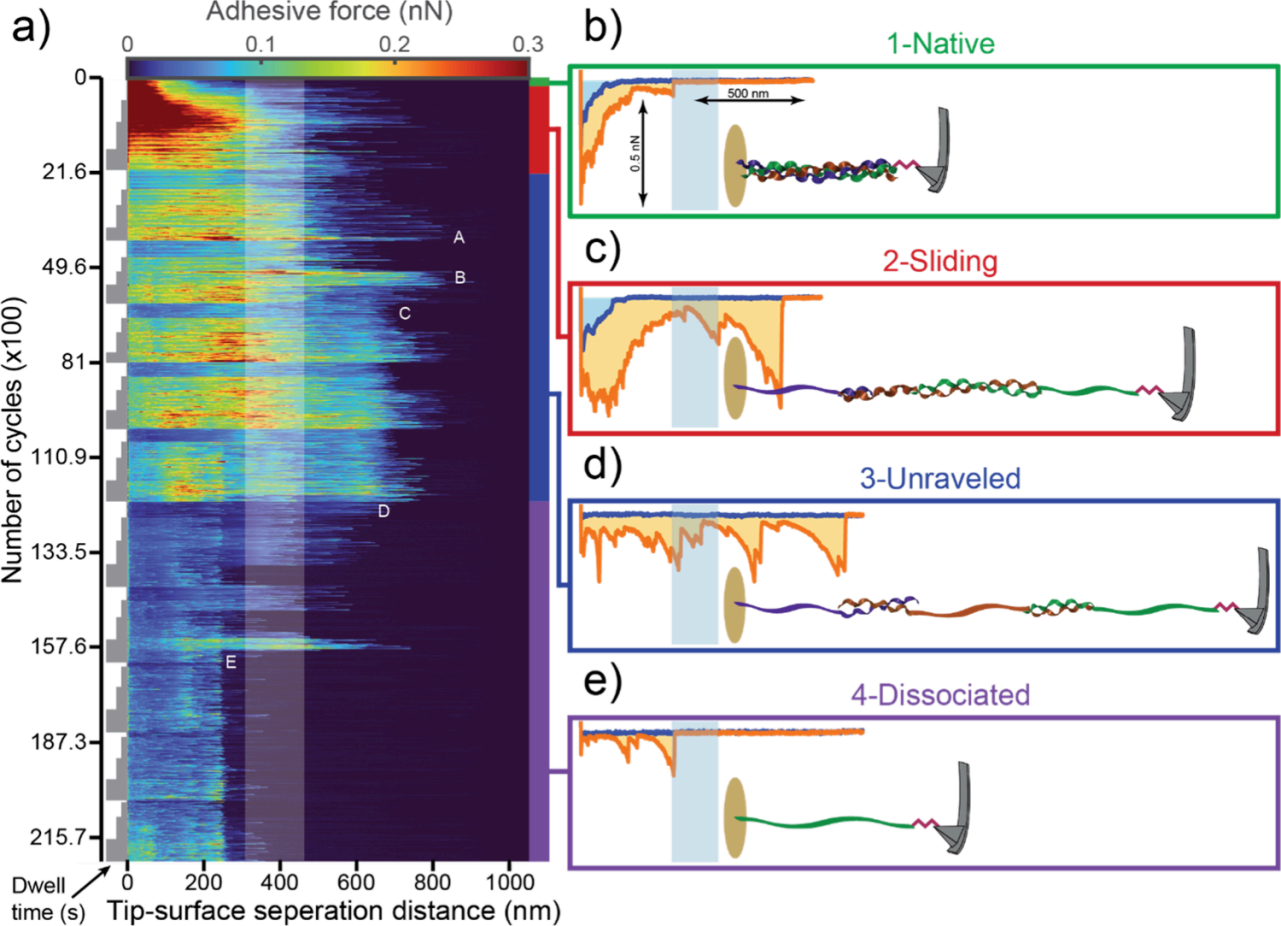
Summary of the force–distance measurements obtained during retraction. (a) Force curve landscape map (FCLM) showing the color-coded adhesive force as a function of the tip–surface distance (*x*-axis) and number of cycles (*y-*axis). Each horizontal line in the image shows the average of eight consecutive force–distance cycles (see SI for details). The shaded vertical band indicates the range of lengths calculated for the TC-linker complex. The colored bars to the right of the image mark the different stages of TC denaturation caused by stretching. The gray boxes to the left indicate the dwell times (0s, 1s, 2s, 4s). Notable events are labeled (A, B, C, D, E). (b–e) Examples of force–distance curves at different stages of TC denaturation, from the native state (green) to the sliding phase (red), unraveled state (blue), and dissociated state (violet). The illustrations show the molecular conformation immediately before pull-off.

#### Native State

In the first 130 cycles (green bar in Figures 3a and 4c), the pull-off length ranged between 167 and 454 nm and did not exceed the range of lengths (308–463) nm calculated for the TC-linker complex (see ″Length of the TC-linker complex″ in SI). At short distances (>20 nm), adhesive forces in the order of 0.5 nN were found (Figure 3a), likely due to electrostatic attraction between the negative mica surface and positive amino groups that remained from the amino-functionalization step (Figure S2b). These interactions were followed at larger distances by weaker adhesive peaks of the order of 0.4 nN (Figure 4b). The average work of adhesion was distributed in the range (30–40)×10^–18^ J, approximately equal to 10^4^ *k*_*B*_ *T* (Boltzmann thermal energy) (Figure 4d). This behavior indicates that the TC was initially in the native state and deformed reversibly in response to consecutive cycles.

**Figure 4:**
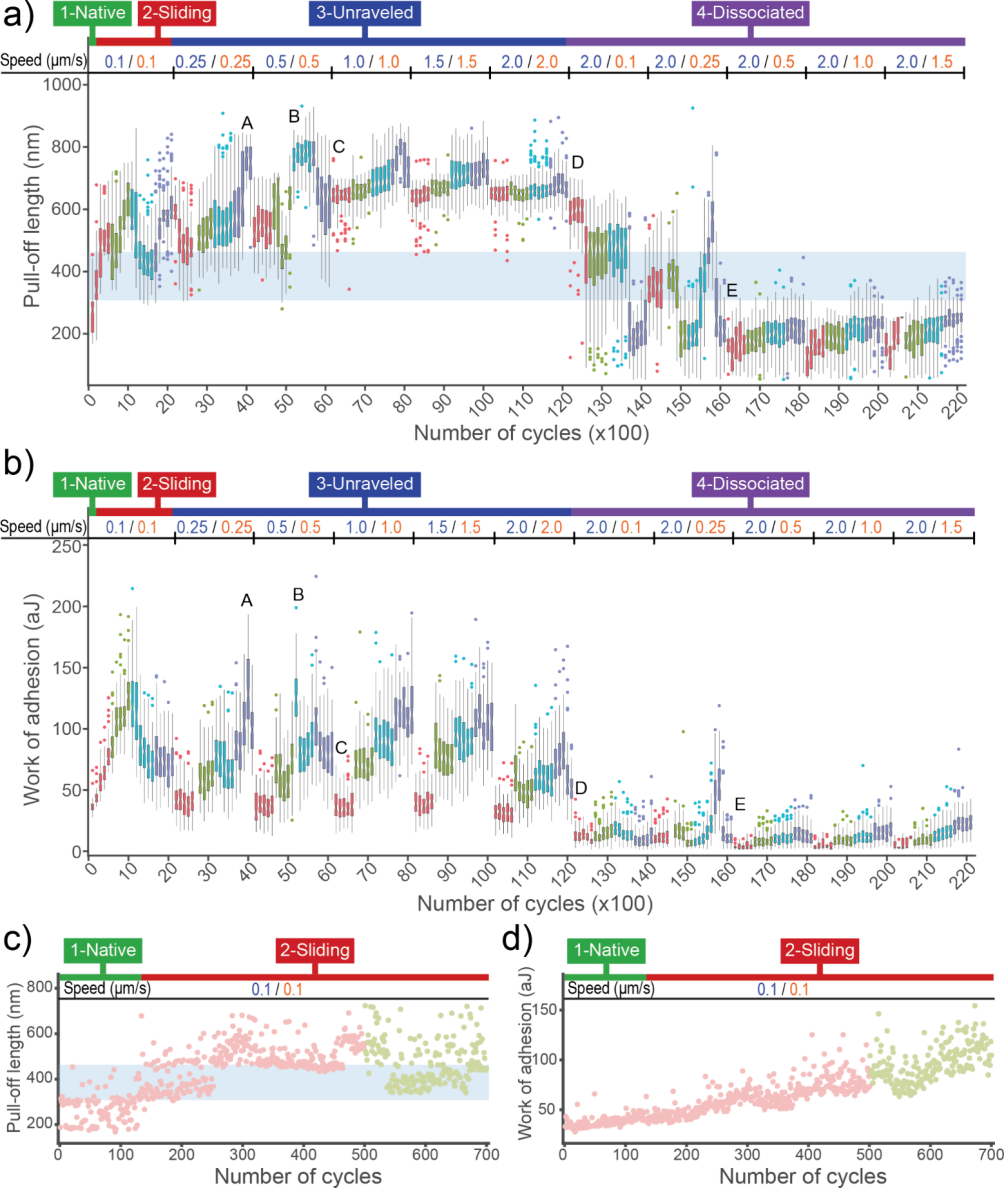
Data of Figure 3 grouped in sets of 100 cycles, corresponding to the 10 × 10-point grid of Figure 1c. (a) Pull-off length *L*_*pull-off*_. The blue vertical band indicates the calculated length range of the TC-linker complex. (b) Work of adhesion *W_adhesion_*. The boxplots extend from the lower to the upper quartile, *i.e.*, comprising half the data set. The whiskers go from minimum to maximum and dots indicate outliers. (c, d) Enlarged view of first 700 cycles. The approach/retraction speeds are indicated as pairs of blue/orange numbers. Different stages of TC denaturation are indicated by colored stripes and labeled boxes (native state in green, sliding phase in red, unraveled states in blue, and dissociated state in violet). The color of the boxplot in (a) and (b) and dots in (c) and (d) indicates the dwell time: red -0s, green -1s, emerald -2s, and magenta -4s. Figure created using gramm data visualization toolbox.^42^

#### Sliding Phase

After about 130 cycles, the average pull-off length rapidly increased above the maximum theoretical length of the TC-linker complex, with single measurements reaching as much as 800 nm. Clearly, the TC unraveled and denatured because it was stretched beyond the elastic limit of the native triple-helix structure. Most likely, the α-chains slid relative to each other within the TC, as shown schematically in Figure 3c. In this sliding phase (red bar in Figure 3a, Figure 4, Figure S4), the pull-off length varied from one cycle to the next much more than in the native state, and the average value oscillated above the TC-linker complex length (Figure 4a). Moreover, the molecules did not show a consistent response to changes in retraction speed or dwell time (Figure 4a). Additionally, the attractive forces were constantly evolving during tip–surface approach, reaching 0.2 nN with snap-in lengths over 100 nm before weakening again and finally disappearing completely (Figure S4). This variability in both approach and retraction indicates that the unraveled TC did not recover the native state after pull-off and did not form a stable state with well-defined length and response to stretching.

The adhesion forces (Figure 3a) and work of adhesion (Figure 4b) followed a similar trend as the pull-off length (Figure 4a), interpreted as unraveling and elongation of TC. In this process, TC also became more flexible in regions comprising only one or two α-chains (Figure 3c). Therefore, the molecule could flatten over a larger area in contact with the mica surface, increasing the number of surface bonds and their contribution to the work of adhesion. In addition, unraveling exposed amino acids that were initially hidden within the native triple-helix structure. These amino acids could form new surface bonds, notably hydrogen bonds with siloxane groups at the mica surface,^23,24^ contributing to the work of adhesion. Furthermore, unraveling and elongating the TC required breaking numerous hydrogen bonds between the α-chains, adding to the total work of adhesion.

#### Unraveled Dimer and Trimer States

After about 2000 cycles, the response to changes in dwell time became more consistent and reproducible (blue bar in Figure 3a and Figure 4a-b). The average pull-off length was in the range of (470–550) nm for short dwell times but tended to randomly jump to about 750 nm for longer dwell times (points A and B in Figure 4a). As the number of cycles increased, the average pull-off length increased and stabilized in the relatively narrow range of (670–820) nm, with large values corresponding to longer dwell times (starting from point C in Figure 4a). This behavior suggests that the TC reached the ultimate state of unraveling, *i.e.*, a fully stretched yet stable and unbroken trimer with chains overlapping at their ends (Figure 3d). Such unraveled state either persisted (without further chain sliding) or was reproduced (after sliding the chains back together) over thousands of cycles. In this regime, both the average work of adhesion and pull-off length increased consistently with the dwell time because the number of tip–surface interactions increased, resulting in a longer pulling time and pull-off length. The fully unraveled state occasionally produced pull-off lengths as large as 900 nm (Figure 3a, Figure 4a), approximately equal to three times the length of an α-chain (see ″Length of the TC-linker complex″ in SI).

From an energy perspective, the work required to slide one α-chain out of a TC can be estimated and compared to the results shown in Figures 3 and 4. The work of adhesion *W_adhesion_* (Figure 1e and Figure 4b and 4d) is the mechanical work done on the TC molecule to break the bonds between α-chains and unravel the triple helix, slide and stretch the α-chains, and peel the molecule from the substrate. While it is not possible to determine how much of the work of adhesion *W_adhesion_* goes into each of these processes, the energy input required to break the hydrogen bonds between α-chains can be estimated. Based on a peptide model,^26^ we estimated the backbone amide protons of each Glycine residue form an interchain hydrogen bond with the carbonyl oxygen of the X residue in the adjacent chain. Consequently, there are about 333 Glycine residues among the approximately 1000 amino acids of an α-chain^27^ forming interchain hydrogen bonds with the X residues of the other α-chains in the TC triple helix. In addition, approximately another 333 hydrogen bonds are formed from the Glycine residues of these two α-chains with the X residues of the first α-chain. Therefore, there are about 670 hydrogen bonds in total. The energy required to break a hydrogen bond is (4–30) kJ/mol,^28–30^ or (0.007–0.05 aJ) at room temperature, corresponding to (2–8)*k*_*B*_ *T*. Therefore, a total energy of (5–34) aJ is required to break 670 hydrogen bonds. The work of adhesion in the experiments was *W*_*adhesion*_ = (10–225 aJ), with a mean of (69 ± 30) aJ, in the sliding phase and unraveled states (corresponding to almost 7000 cycles, Figure 4b and 4d). These values suggest the presence of more than sufficient energy to break all hydrogen bonds within the TC triple helix. An additional consideration is whether the forces in the experiments were large enough to initiate sliding and unraveling. Figure 2a shows that the force measured during retraction exceeded 600 pN with peak values reaching 1400 pN. These forces were large enough to break a single hydrogen bond, *i.e.*, a bond with a length of 0.3 nm and a force of (23–170) pN. Moreover, in MD simulations that modeled the sliding and unravelling of the TC molecule as a whole,^14^ the ultimate force experienced during sliding was reported to be around 500 pN. Therefore, our heuristic estimates suggest that both energy and force criteria were fulfilled for unravelling the TC and sliding of α-chains.

#### Dissociated State

Eventually, the unraveled trimer dissociated by losing one or more α-chains to the mica surface (point D and violet bar in Figure 3a and Figure 4a-b). Indeed, both the average work of adhesion and pull-off length suddenly decreased after about 12500 cycles. Although values as large as 750 nm were obtained for up to 15900 cycles, the pull-off length was most frequently found to be around 580 or 250 nm, ultimately stabilizing in a very narrow range around the latter value (point E in Figure 3a and Figure 4a). These two values of *L_pull‑off_* correspond to the fully stretched length of two α-chains overlapping at their ends (dimer) and an individual α-chain (monomer), respectively. Larger values were obtained occasionally by picking up and temporarily reassociating both α-chains that were previously lost to the surface until these chains became firmly adsorbed to the surface.

The adhesive peaks obtained in the fully dissociated monomer state (Figure 3e) showed the typical force–distance dependence expected for the pulling of an end-tethered peptide chain, *i.e.*, an α-chain grafted to the AFM tip. Indeed, recent AFM measurements on individual molecules of denatured collagen (gelatin) show that the adhesive force *F* increases as a function of tip–surface distance *D* according to the freely jointed chain model:

$$D=L\left(1+\frac{F}{ak}\right)\left[\coth\left(\frac{aF}{k_{B}T}\right)-\frac{k_{B}T}{aF}\right]$$ 1

where *a* = 0.7 nm is the Kuhn segment length, *k* = 14 nN/nm is the stretching stiffness of the Kuhn segment, *L* = *Na* is the contour length of the chain portion being stretched, comprising *N* segments, and *k*_*B*_ *T* is the Boltzmann energy.^31^ Figure 5a shows that this model also agrees with the force-curve obtained in the fully dissociated state of TC with Kuhn lengths *a* ranging between 0.4 and 0.9 nm, comparable with the size of a single amino acid (*e.g.*, distance of 0.4 nm between consecutive α-carbons), and contour lengths *L* between 150 and 250 nm, consistent with the length of an α-chain. This finding confirms that the fully dissociated state is a single peptide chain. In comparison, the triple-helix structure of native TC has a persistence length larger than 10 nm^32^ and, in our experiment, produced force–distance curves that were qualitatively different from the ones predicted for a stretched freely jointed chain (Figure 3b).

**Figure 5:**
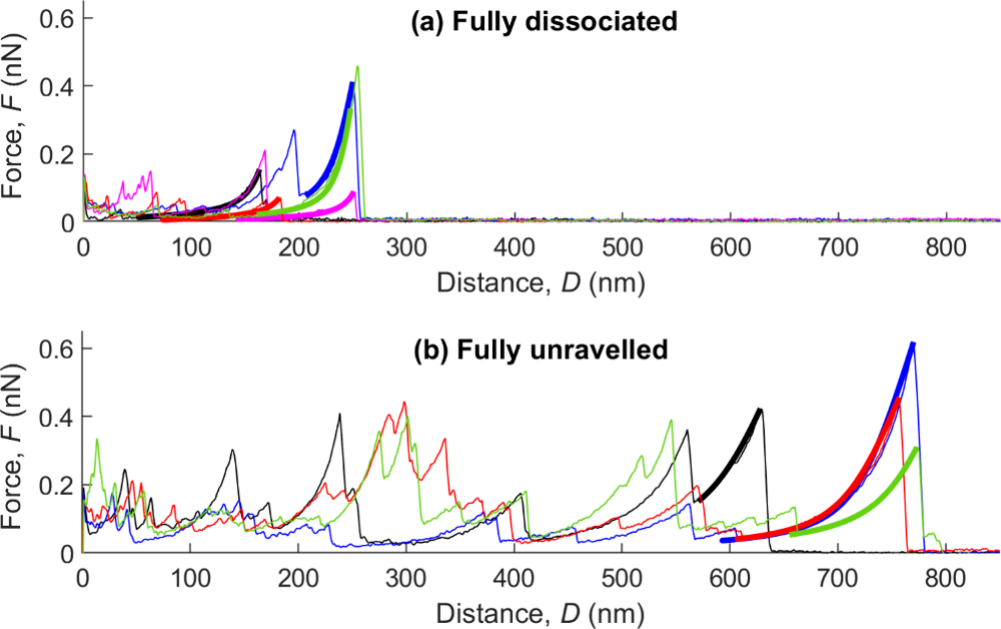
Adhesive force *F* as a function of the tip–surface separation distance *D* in (a) the fully dissociated and (b) fully unraveled state. Thin and thick lines are experimental data and fits to the free jointed chain model (eq 1), respectively, with Kuhn length *a* ranging between 0.4 and 0.8 nm, variable contour length *L*, and constant segment elasticity *k* = 15 nN/nm equal to the value measured for gelatin.^31^

Interestingly, force peaks accumulated at distances of 250 and 580 nm even before dissociation (Figure 3a), indicating that the unraveled trimer and dimer adhered to the mica surface more strongly at the overlapping chain segments. Moreover, the final adhesive peak measured in the fully unraveled trimer state before dissociation also agreed with the freely jointed chain model, confirming that a single peptide chain occupied most of the trimer length (Figure 5b).

Most likely, the sudden drop in the work of adhesion after the first dissociation event (point D in Figure 3a and Figure 4a-b) was due to the dissociated α-chains being adsorbed on the mica surface, where they created a repulsive electrostatic background against the α-chains still attached to the AFM tip. Note that the first dissociation occurred shortly before decreasing the retraction speed. After this event, the work of adhesion never recovered the values obtained before dissociation, even after decreasing the retraction speed or increasing the dwell time.

Remarkably, the first three states/phases (native state, sliding phase, unraveled trimer and dimer states) lasted for about 15900 cycles before complete dissociation. Such exceptional toughness of TC, maintaining cohesion despite irreversible structural rearrangement, has direct relevance to the damage tolerance and resilience of collagen fibrils and collagen-rich tissues. Notably, ultimate tensile strains of 35% and more have been reported for collagen fibrils,^3^ particularly those having a sufficient number of enzymatic or nonenzymatic cross-links.^33^ MD and coarse-grained models connect such high strains to bond-breaking and mechanical denaturation (sliding phase and unraveled state) of TC.^14,34^ Our findings provide experimental support for these model predictions and help explain how collagen fibrils are able to deform to high strain and achieve high toughness, which in turn also translates to larger structures in tissue hierarchy such as tendon fascicles and whole tendons.

### Limitations

Our study has a few limitations to be addressed. First, type-III collagen is less abundant than type I, which is the most abundant one. Second, type-I TC does not contain cysteine and, therefore, is not suitable for the bioconjugation protocol used in this study. Nevertheless, type-III TC bears a very similar triple-helix structure compared to type I, importantly the Glycine-X-Y amino acid sequence, suggesting similar mechanical behavior and deformation mechanisms. Third, the experiments reported here were not carried out at physiological conditions of near-neutral pH and relatively high salinity, where collagen normally serves its structural and mechanical functions and, therefore, is expected to be most robust. As explained in the SI (″Choice of media and substrate surface″), only mica and acidic solution produced strong and reproducible TC-substrate adhesion (cf. Figure 2b), revealing the surprisingly high toughness of the collagen molecule. We propose that the mechanism underlying this toughness, *i.e.*, α-chains sliding alongside and eventually ″catching″ each other by their ends, contributes to the material toughness observed at larger length scales in collagen tissues. Therefore, it is unlikely that the molecular mechanism of chain sliding and catching disappears at physiological conditions. On the contrary, one may expect TC to be even tougher against the unravelling of the triple helix at the physiological condition where it normally functions. Finally, type-III TC is homotrimeric and each α-chain has two cysteine residues providing a total of six possible conjugation sites. We do not know at which site the linker attaches to the TC. This suggests the possibility that several linkers could be attached to one molecule or that there are multiple molecules attached to the tip and interacting with the substrate. For the bioconjugation protocol used here, we have estimated an average of 1.5 linkers to the AFM tip apex, and we have seen double conjugation only in about 10% of the conjugate tips.^17^ In addition, the linkers are large compared to the distance between α-chains at the end of the TC. It is therefore unlikely that more than one α-chain was attached to the AFM tip via multiple linkers. Crucially, this conclusion is verified *a posteriori* by the AFM force measurements, where no outstanding large-force events were observed that might have revealed the rupture of a disulfide bond as the TC unraveled. Further, if two α-chains were attached to the tip surface via disulfide bonds, one of these bonds should have ruptured to reach the fully unraveled trimer or dissociated states, producing a single large-force peak. Instead, the fully dissociated state was reached through an irregular sequence of small-force peaks, consistent with a single α-chain permanently grafted to the AFM tip via an unbroken disulfide bond.

## Conclusions

Our findings provide direct experimental evidence of TC denaturation under stretching. Driven by multiple electrostatic bonds between TC and mica surface, which sequentially broke, the triple helix unravels and eventually dissociates under cyclic stretching. *In vivo* and *ex vivo* studies have reported a similar degradation process in tissues where shear load was transmitted and controlled between TCs using collagen-hybridizing cross-linking peptides.^14,35^ In contrast, the sliding of the α-chains in our experiments involved breaking the native bonds between α-chains within a TC. Indeed, pulling one α-chain from one end of TC triggered chain sliding (Figure 2b-c), whereas previous AFM studies on TC with random pulling geometry did not identify this process.^36,37^

The exceptional toughness of individual TC, withstanding stretching ratios as high as 2.5 without breaking for over 15900 cycles, agrees well with the ultimate strain predicted by MD simulations of TCs stretched by their ends.^14^ In addition to MD simulations, our experiments show the exceptional resilience of TCs to cyclic loading and highlight the connection between the high damage tolerance of collagen-rich tissues and the toughness of collagen molecules. The sliding of α-chains within the TC releases conformational hidden segments of the native collagen triple helix by sacrificing intramolecular bonds so as to distribute and sustain an applied tensile force over a more extended protein structure. Similar molecular mechanisms have been found in other proteinaceous materials serving the critical biomechanical role of sustaining large tensile loads and preventing fracture propagation within collagenous tissue, such as bones^38^ and muscles.^39^ Moreover, completely or partly unraveled α-chains are longer and more flexible and interact with their neighbors in an expanded interaction volume. Therefore, we argue that TC unraveling facilitates intermolecular cohesion and, to some extent, compensates for the loss of intramolecular order. Under physiological conditions, intermolecular cross-linking is likely required to trigger α-chain sliding and pull-out under intense shear loading.^14^ Importantly, the remarkable resilience of collagen fibrils and collagen-rich tissue is a desirable property for biological organisms, as it ensures remaining cohesion over large lengths as a recovering mechanism from traumatic overloads.

## Materials and Methods

### AFM Probe Tip-Tropocollagen Bioconjugation Protocol

Individual type-III tropocollagens (TCs) were covalently attached to the silicon tip of an AFM probe (MSNL-10 from Bruker, Germany) by adapting an established bioconjugation protocol^40^ with minor modifications. The AFM probe surface was oxidized by exposure to air for at least 30 min, washed in chloroform (3 × 5 min), and dried in a gentle stream of nitrogen to create a thin layer of silicon oxide and silanol groups.

For the amino-functionalization of the AFM probe tip, 4.95 mg ethanolamine hydrochloride (from TCI Deutschland, Eschborn, Germany) was dissolved in 9.9 mL dimethyl sulfoxide (DMSO), heated to *ca.* 70 °C, to obtain a concentration of 0.5 mg/mL. The solution was cooled and degassed for 30 min in the presence of molecular sieves (0.4 nm) by applying a vacuum (15 min at 100 mbar followed by 15 min at 10 mbar). The AFM probe was immersed in this solution and incubated overnight. The following day, the probes were washed with DMSO (3 × 1 min) and ethanol (3 × 1 min) and dried in a gentle stream of nitrogen.

In the next step, the linker was attached. Therefore, 12 mg of the thiol linker, a maleimidopropionyl-polyethylene glycol-hydroxysuccinimide ester (MI-PEG-NHS) with an average of 27 ethylene glycol units (from Polypure AS, Oslo, Norway), was dissolved in 2 mL chloroform to obtain a concentration of 6 mg/L and supplemented with 120 μL triethylamine. The AFM probe was placed into custom-built Teflon PTFE (Polytetrafluorethylen) reaction chambers filled with linker reaction solution and was incubated overnight. The following day, the AFM probe was washed with chloroform (3 × 10 min) and dried in a gentle stream of nitrogen.

To attach the TC, a volume of 0.5 mL of TC solution, dissolved in HCl (3.4 mg/mL, product no. ab73160, lot no. GR104699–42 from ABCAM, Cambridge, UK) was diluted in 4.5 mL of ultrapure water (18.2 MΩ·cm resistivity from Milli-Q, Merck, Germany) to obtain a concentration of 0.34 mg/mL. A 2 mL volume of this solution was then mixed with 40 μL ethylenediamine tetraacetic acid (EDTA), 100 μL 4-(2-hydroxyethyl)-1-piperazine-ethane-sulfonic acid (HEPES) and 40 μL tris­(2-carboxyethyl)­phosphine (TCEP, from TCI Deutschland, Eschborn, Germany). We monitored pH and temperature after each step and used HEPES to adjust to pH 7.6. Subsequently, 500 μL of this solution was pipetted onto the AFM probe, which was placed in the custom-built Teflon PTFE reaction chambers and incubated overnight, resulting in site-specific bioconjugation of type-III collagen at position 1196 or 1197 (two neighboring cysteine residues) of one α-chain.

The following day, the AFM probes were washed with sterile-filtrated 0.5 mol/L acetic acid (1 × 5 min) and sterile water (2 × 5 min). The functionalized probe was placed into a custom-built Teflon PTFE box filled with sterile water used for transport and storage.

### AFM Force Measurements Protocol

AFM experiments were performed using a NanoWizard ULTRA Speed AFM system (JPK Instruments AG, Berlin) equipped with an inverted optical microscope (Axio Observer D1, ZEISS) and placed on a Halcyonics i4 vibration isolation system (Accurion, Göttingen, Germany). The whole system was located in an acoustic enclosure placed on a stable base (JPK, Berlin, Germany). The AFM cantilever (Bruker MSNL10, Cantilever E) had a triangular shape with 0.1 N/m nominal spring constant, and the nominal tip radius was 2 nm.

Nonfunctionalized AFM probes were thoroughly investigated using an inverted light microscope to ensure that only clean and intact AFM probes were used for force measurements. These probes were cleaned in chloroform, and the cantilever spring constants were determined using the thermal vibration method in air.^41^ The mica substrate was glued onto the bottom part of a glass Petri dish using UV glue and hardened in an UV-curing machine (405 nm wavelength from Sovol 3D). Both the tip and substrate were immersed in an aqueous solution in a clean Petri dish and thoroughly rinsed with the solution before any experiment. All AFM measurements were obtained in the (28–31)°C temperature range.

To calibrate the force–distance measurements, the tip was pushed onto a stiff surface of freshly cleaved mica in acetic acid until the cantilever deflection varied linearly with the surface displacement. In this ″hard-wall″ regime, the tip–surface separation distance was taken as zero, and the cantilever deflection was equal to the surface displacement. Force curves with TC-functionalized cantilevers where recorded in acetic acid soluton at pH 3.5 on mica surfaces. Approach and retraction speeds as well as surface dwell times were varied, and for each combination 500 force curves were recorded on a 10 × 10-point square grid with 10 × 10 μm^2^ surface area. The specific approach and retraction speeds used were 0.1, 0.25, 0.5, 1.0, 1.5, and 2.0 μm/s and the dwell times were 0, 1, 2, and 4 s.

### Data Analysis

For the analysis of the AFM force–distance measurements, a custom-built MATLAB script (2021b, The MathWorks Inc., Natick, Massachusetts, USA) was used.

#### Data Preprocessing

The cantilever sensitivity was determined for each curve, and data artifacts caused by laser interference were corrected by applying a correction fit to the noncontact region of the force–distance data. Also, any slope in the force–distance curve in this region was corrected by applying a linear fit. Then, the tip–substrate distance was determined assuming that the tip and substrate were incompressible, i.e., a hard-wall repulsion with zero distance was eventually reached under a sufficiently larger compressive load. Corrupted force curves due to laser artifacts or missing data points were excluded by visual examination.

#### Force Curve Selection Procedure

To ensure a proper analysis, the remaining force curves were further examined to identify those that showed TC adhesion events. Therefore, predefined thresholds for the approach and retraction part needed to be surpassed. To detect snap-in behavior and distinguish from background noise, a threshold of 15 pN adhesive force had to be surpassed in the approach part of the curve from 200 nm to the contact point. Similarly, a threshold of 25 pN had to be surpassed in the retraction part between 20 and 300 nm to detect TC adhesion events. The reason to start not at the contact point, but rather at 20 nm from it, was to exclude force peaks caused by amino groups on the AFM probe within *ca.* 10 nm from the surface (see Figure S2b,c). These amino groups are remnants from the amino-functionalization step of the bioconjugation process.

#### Force Curve Feature Detection

The remaining, selected force curves were processed to determine various features, *i.e.*, snap-in length (introduced in Figure S4), pull-off length, maximum adhesion force, maximum attractive force, and work of adhesion. Although the automated feature detection of the script was reliable, it was occasionally unsatisfactory. Force curves with badly detected features were excluded from further data processing by visual examination. The data shown in Figure 2, Figure 3, and Figure S4 originate from an experimental run where 74% (16429 out of 22100) of the detected force curves could be analyzed.

#### Force Curve Landscape Maps (FCLMs)

A FCLM is a graphical, two-dimensional representation of the force–distance curves obtained during approach or retraction as a function of the cycle number. In detail, the color-coded adhesive force is presented as a function of the tip–surface distance (*x*-axis) and number of cycles (*y-*axis). Data processing was needed to produce the FCLMs of the approach (Figure S4) and retraction part (Figure 2a) shown. First, only force curves that passed the selection procedure (see ″*Force curve selection procedure″*) were admitted for selection. For the retraction FCLM, a total of 16429 force curves (whole experimental run) were selected, whereas only the first 2441 force curves for the approach FCLM were selected. After applying a rolling average filter, the selected data was down-sampled to fulfill the resolution requirements of the FCLMs (512 × 512 pixels for the approach FCLM, 1024 × 2048 pixels for the retraction FCLM).

## Supplementary Material



## Notes

Length of the TC-linker complex, Choice of medium and substrate surface, Verification of a successful bioconjugation, AFM measurements of adsorbed TC layers, SFA force–distance measurements; Figure S1: Crystal structure-based collagen α-chain sequence; Figure S2: Representative force–distance curves; Figure S3: Optimal solvent and surface combination for the AFM SM experiments; Figure S4: Summary of the force–distance measurements obtained during approach; Figure S5: AFM imaging of adsorbed layers of TC; Table S1: Calculated backbone lengths; Table S2: AFM force spectroscopy derived linker length; SI References (PDF)
