# Supplementary material for: Unraveling and Sliding of Polypeptide Strands Underlies the Exceptional Toughness of the Triple-Helix Collagen Molecule
Source: ACS Nano. 2026 Jan 9;20(3):2730–9. doi: 10.1021/acsnano.5c15873 (PMC12854733; doi:10.1021/acsnano.5c15873)
Supplement: Supplementary file 1 [file nn5c15873_si_001.pdf]

Supporting Information for

# Unraveling and sliding of polypeptide strands underlies the exceptional toughness of the triple-helix collagen molecule

*Andreas Rohatschek[1,6,7], Bruno Zappone[2,*], Patrick Steinbauer[3,4,7], Manuel Rufin[1,7], Daniela A. Barragan Rivera[2,5], Maria P. De Santo[5], Orestis G. Andriotis[1,7], Stefan Baudis[3,4,6,7] and Philipp J. Thurner[1,6,7,*]*

[1] Institute of Lightweight Design and Structural Biomechanics, TU Wien; Gumpendorfer Straße 7 / Objekt 8, 1060 Vienna, Austria.

[2] Consiglio Nazionale delle Ricerche - Istituto di Nanotecnologia (CNR-Nanotec); Via P. Bucci 33/C, 87036 Rende (CS), Italy.

[3] Institute of Applied Synthetic Chemistry, Division of Macromolecular Chemistry, TU Wien; 1060 Vienna, Austria.

[4] Christian Doppler Laboratory for Advanced Polymers for Biomaterials and 3D Printing, TU Wien; 1060 Vienna, Austria.

[5] Università della Calabria - Dipartimento di Fisica; Via P. Bucci 31/C, 87036 Rende (CS), Italy

[6] Biointerface Doctoral School, TU Wien; 1060 Vienna, Austria.

[7] Austrian Cluster for Tissue Regeneration; Vienna, Austria.

## Supporting Information Text

### Length of the TC-linker complex

For an adequate analysis and conclusive interpretation of the AFM force spectroscopy results, knowledge about the properties and behaviour of the attached TC-linker complex is of crucial importance. For the AFM experiments performed in this study, the length of the TC-linker complex is the most important property. Although the attached complex consists only of two components – the NHS-PEG-MI linker and TC – determining its length is not a simple task. The actual linker-TC-complex length is depending on several factors, i.e. flexibility, conformational organisation in the medium, attachment points of the linker on the AFM tip and TC on the linker. While some factors are known, i.e. ultrastructure, attachment point of TC on the linker, others are not, i.e. flexibility, attachment point of the linker on the AFM tip.

Taking into account all the different parts influencing the length of the TC-linker complex it is more useful to consider an expected length range rather than an individual length value. To enframe this range, boundary values have to be defined. For the upper boundary value, the contour length of the linker and the TC is suitable since it represents the maximum theoretical length without including molecular conformations. For the lower boundary value experimentally determined linker pull-off lengths combined with TC lengths, derived from crystal structure data, are used.

The contour length of the NHS-$PEG_{27}$-MI linker, calculated by summing up the lengths of all intra-molecular bonds, is 13.47 nm (not shown).

The distance between the α-carbons of two consecutive amino acids is approximately 0.43 nm [1]. Therefore, the contour length of one α-chain calculated by multiplying by the number of amino acids (1068 aa) is 459.9 nm. TC was attached to the thiol linker with a cysteine residue at position 1196 or 1197 near the N-terminus (note that the position count of the collagen type III α-chain [2] starts at the signal peptide which becomes removed, as well as the propeptide, during formation of the native protein). The number of amino acids from the C-terminus to the attached residue was 1043 or 1044, corresponding to a contour length of 449.2 nm and 449.7 nm, respectively.

The total contour length of the TC-linker complex calculated with this method was 463 nm.

The native length of the TC-linker complex can be obtained considering the secondary structure of TC. Four crystal structures of fibrillar collagen sequences displaying the triple helix structure were selected from the world wide protein data bank (PDB) [3]. The backbone length of one α-chain per sequence of each crystal structure was measured in PyMOL [4], a molecular visualization software (see Fig. S1). The length was extrapolated from a 30 aa (amino acid) sequence to the whole α-chain (1068 aa) or to the linker attachment point at position 1044 aa (see Table S1).

A linker length of approx. 16 nm was derived from AFM data by averaging the measured length of 74/1000 (7.4%) force-distance curves (see Table S2).

The resulting average TC-linker complex length was determined with 308 nm.

Based on the performed calculations the expected length range of the TC-linker complex lies within a lower boundary value of 308 nm and an upper boundary value of 463 nm.

## Choice of medium and substrate surface

To achieve strong and reproducible TC-substrate attraction, the optimal experimental test conditions for different media and substrate surface combinations were investigated. AFM cycles in phosphate-buffered saline (PBS) (not shown) and in ultrapure water (Mili-Q®) - see Fig. S3 - showed only very weak interactions on muscovite mica and glass. Measurements in 3-(cyclohexylamino)-1-propanesulfonic acid (CAPS) did not show any interaction. Rinsing with acetic acid instantly and significantly increased the attraction and pull-off length on mica (Fig. S3). Only measurements on mica in acetic acid consistently produced large pull-off lengths, whereas measurements on glass were less reproducible (Fig. S3).

## Verification of a successful bioconjugation

To verify a successful bioconjugation a series of trials were performed. A non-functionalized AFM probe tip as well as each bioconjugation protocol step was investigated individually. AFM cycles were performed under the same conditions – in acetic acid and on a mica surface – as the TC experiments and representative force-distance curves can be seen in Fig. S2. The force-distance curve in Fig. S2a results from a non-functionalized AFM probe tip. The tip-surface interaction was purely repulsive, most likely because the mica and oxidized Si surface of the tip were equally charged. After amino-functionalizing of the tip (Bioconjugation protocol step 1), a single adhesion force peak was measured at a distance smaller than 10 nm both during the tip-surface approach (snap-in) and retraction, most likely due to the electrostatic interaction between the positively charged amino group and the negatively charged mica surface (Fig. S2b). After attaching the thiol-linker (Bioconjugation protocol step 2) onto the AFM probe tip (Fig. S2c) and TC (Bioconjugation

protocol step 3) via the linker (Fig. S2d), multiple adhesive force peaks were observed during retraction with a large variability in pull-off lengths and forces.

**AFM measurements of adsorbed TC layers**

Adsorbed TC layers used in SFA experiments were characterized using an AFM (Nanoprobe from Bruker, USA) in tapping mode (Fig. S5). Recombinant type-III TC (product no. ab73160, Lot. GR104699-42, from Abcam®, UK) was adsorbed on freshly cleaved muscovite mica surfaces from a 0,34 mg/ml TC solution in ultrapure water (18.2 MΩ·cm resistivity from Milli-Q, Merck, Germany) containing hydrochloric acid at pH 3 - 4. The mica surfaces were flat and approximately circular, with a diameter of about 1 cm. They were entirely covered with the solution and sealed in a Petri dish saturated with water vapor to prevent droplet evaporation. After an adsorption time of 2 h, the TC-coated surfaces were abundantly rinsed with ultrapure water and dried with a gentle flow of nitrogen gas.

Topographic images of the adsorbed TC layers were obtained in air using the AFM in tapping mode (Fig. S5). The adsorbed layers were dense and homogeneous, with a root-mean-square (RMS) roughness of about 0.2 nm over a surface area of $2 \times 2$ $\mu m^2$. Notably, AFM images did not show any fiber-like feature related to the triple-helical linear structure of the TC.

**SFA force-distance measurements**

The SFA was used to measure the normal force generated by TC layers adsorbed at the contact between two solid surfaces of mica as a function of the mica-mica separation distance. Details on

the SFA techniques and surface force measurements can be found in seminal publications [5–7]. The surfaces had a cylindrical shape with a 2 cm radius of curvature and were mounted in the SFA with their axes crossed at 90° to ensure a single contact point with a local geometry equivalent to a sphere and a plane (Fig 2–Insets). One of the surfaces was mounted at the free end of a double cantilever spring with an elastic constant of 900 N/m, while the fixed end of the cantilever was displaced with a speed u of a few nm/s, so as to increase or decrease the surface separation distance. Type-III TC was adsorbed on mica from a 0.34 mg/ml aqueous solution containing hydrochloric acid (HCl) with pH (3.0 – 3.5). The adsorbed TC layer was pressed against either an uncoated mica or another TC-coated surface, either in the same HCl solution or after rinsing the surfaces with an acetic acid ($CH_3COOH$) solution at the same pH. The Debye length ($\lambda$) of such an 1:1 acid electrolyte solution lies between 9 – 17 nm [5].

**Table S1.** Calculated backbone lengths based on the crystal structures 1qsu [8], 1bkv [9], 6a0a [10], and 1k6f [11] from the RSCB protein data bank.

| PDB ID | Sequence length (aa) | Backbone length (nm) | Extrapolated length for 1068 aa (nm) | Extrapolated length for 1044 aa (nm) |
|---|---|---|---|---|
| **1qsu** | 30 | 8.58 | 305.45 | 298.58 |
| **1bkv** | 30 | 8.41 | 299.39 | 292.67 |
| **6a0a** | 30 | 8.34 | 296.90 | 290.23 |
| **1k6f** | 30 | 8.23 | 292.98 | 286.40 |
| | **Mean** | **8.39** | **298.68** | **291.97** |

**Table S2.** AFM force spectroscopy derived linker length of the NHS-$PEG_{27}$-MI linker. Two different speeds were used and a total of 1000 cycles were performed.

| Experiments | Speed | | Cycles | | Linker length | | |
|---|---|---|---|---|---|---|---|
| | Approach | Retraction | Total | Analyzable | Mean | Min | Max |
| | (µm/s) | | (1) | | (nm) | | |
| **1** | 0.5 | 0.5 | 200 | 20 | 17.87 | 10.61 | 38.34 |
| **2** | 2.0 | 2.0 | 800 | 54 | 15.72 | 10.01 | 44.39 |
| | | **Total** | **1000** | **74** | **16.30** | | |

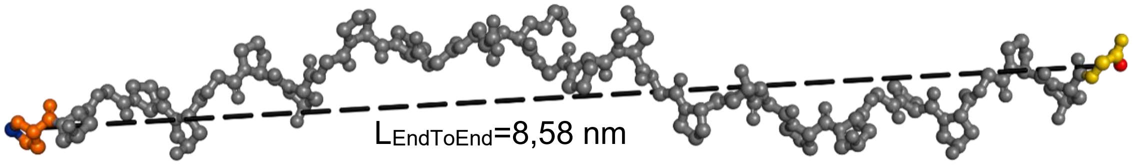


**Fig. S1.** Crystal structure-based collagen α-chain sequence with a length of 30 aa. The end to end length of 8.58 nm was calculated with PyMol using the PDB dataset 1qsu[8]. The colors highlight the terminal regions: N-terminus in orange, C-terminus in yellow, N-terminal nitrogen atom in blue, and the C-terminal oxygen atom in red.

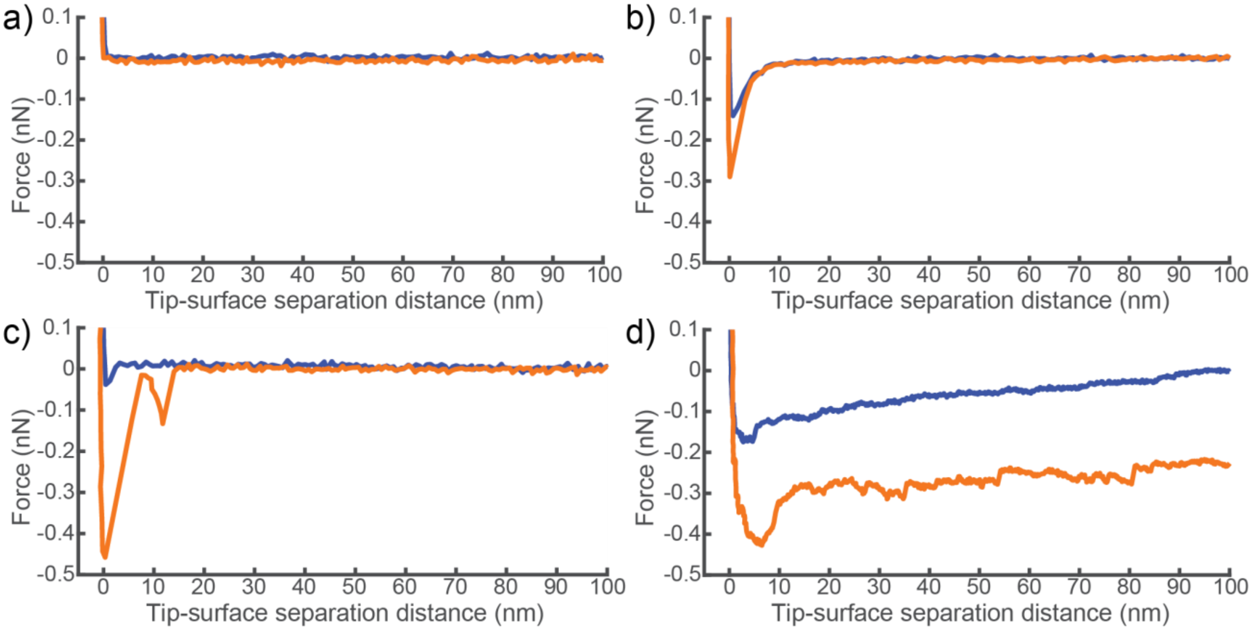


**Fig. S2.** Representative force-distance curves obtained at different steps of the bioconjugation procedure. All measurements were done in acetic acid solution on freshly cleaved mica surfaces. Approach and retraction are shown in blue and orange, respectively. (a) Non-functionalized AFM tip. (b) Amino-functionalized tip. (c) Linker covalently attached to the tip. (d) TC covalently attached via the linker.

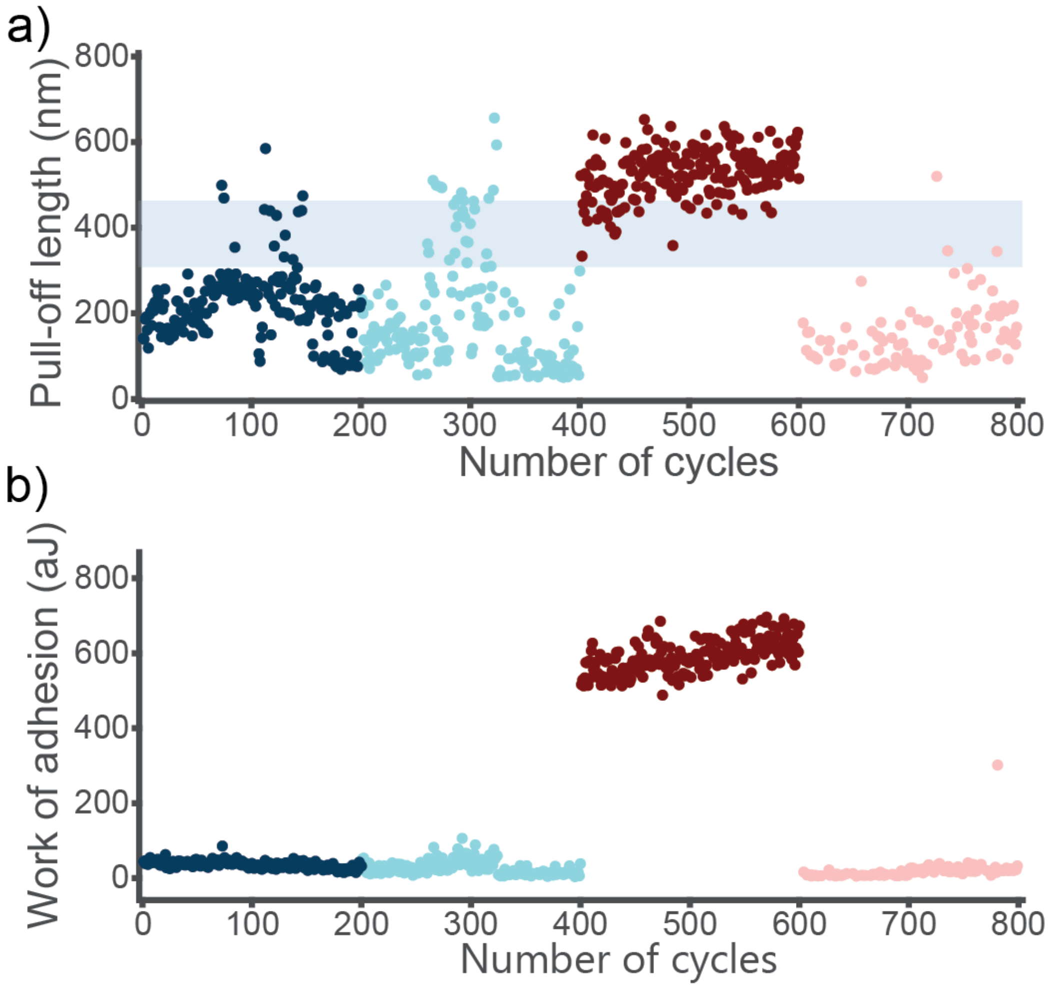


**Fig. S3.** Identifying the optimal solvent and surface combination for the AFM SM experiments. a) Pull-off lengths and b) Work of adhesion measured chronologically for 800 cycles in different media (acetic acid, water) and on different substrates (glass, mica). Acetic acid in red and water in blue. Glass in light hue and mica in dark hue. Each colored dot represents a cycle. The blue band represents the expected TC-linker complex length.

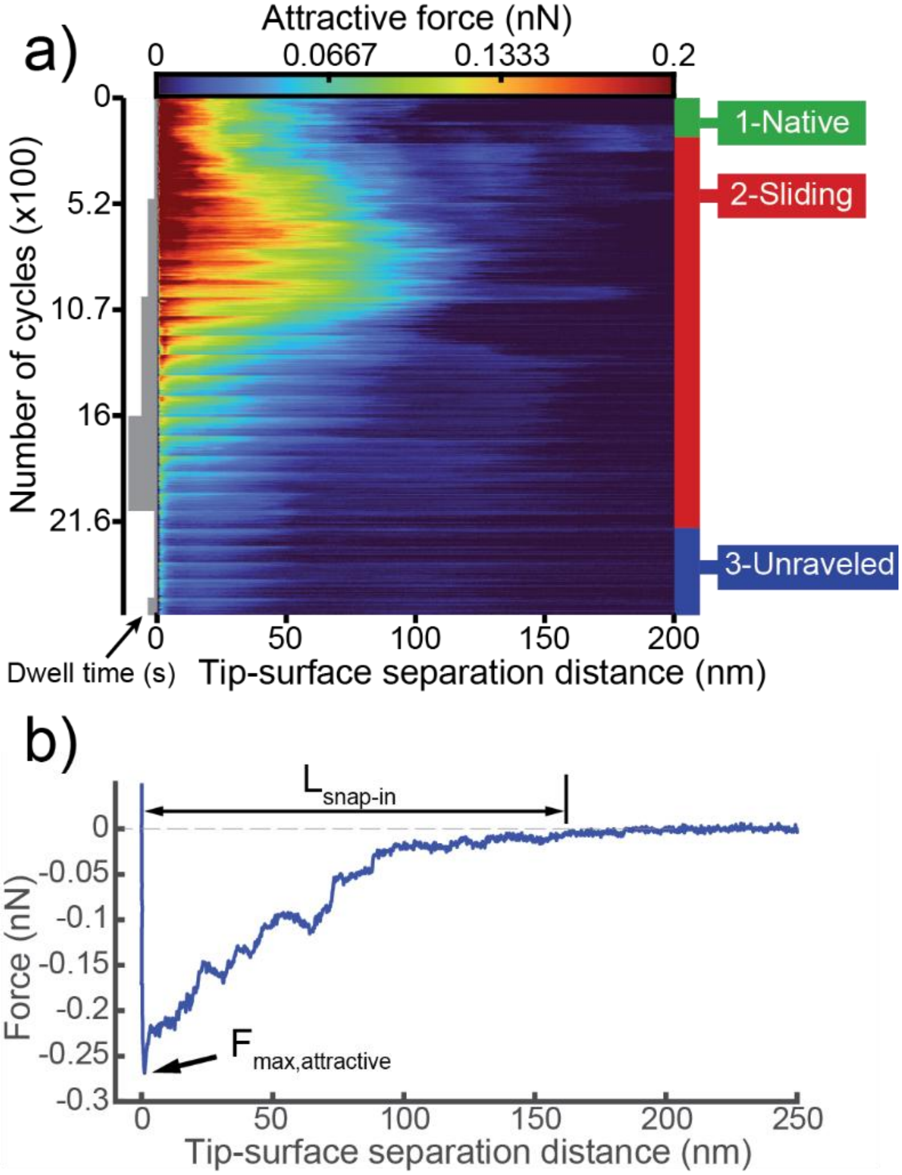


**Fig. S4.** Summary of the force-distance measurements obtained during approach. a) Force curve landscape map (FCLM ) showing the color-coded attractive force as a function of the tip-surface distance (x-axis) and number of cycles (y-axis). Each horizontal line in the image shows the force-distance curve averaged over five consecutive approach measurements. The colored bars to the right of the image figure mark the different stages of TC denaturation caused by stretching. The gray boxes to the left indicate the dwell time (0s, 1s, 2s, 4s). b) Example of a force-distance curve showing only a selection of the approach part detected during one AFM measurement cycle. The measured force (y-axis) is presented as a function of the tip-surface separation distance (x-axis).

The determined parameters are snap-in length ($L_{snap\text{-}in}$) and maximum attractive force ($F_{max,attractive}$).

The blue line represents the approach part of one cycle.

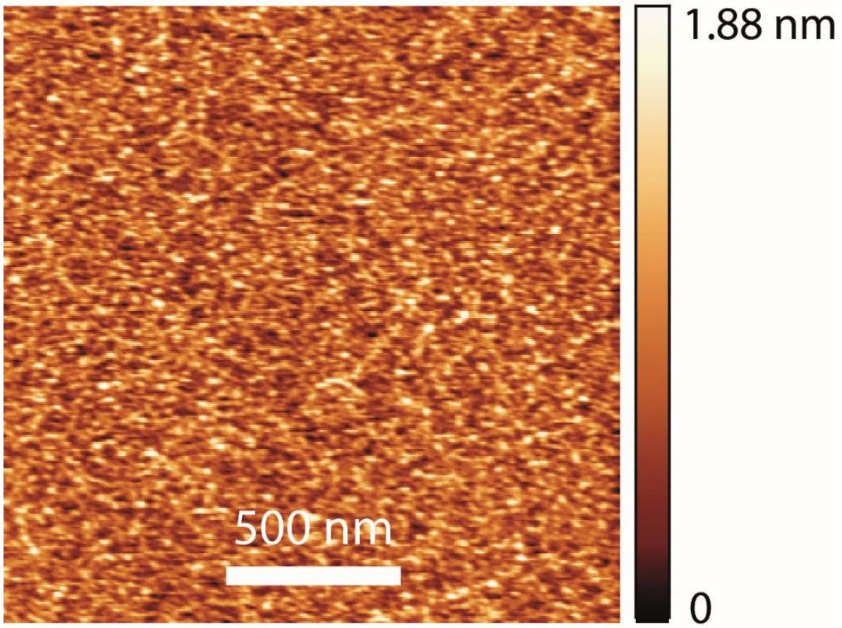

**Fig. S5.** AFM image showing the topography of an adsorbed layer of TC type III in air.

**SI References**


(1) Bond Lengths in Crystalline Organic Compounds. In *CRC Handbook of Chemistry and Physics*; Haynes, William M., N. I. of S. and T., Ed.; CRC Press/Taylor and Francis, Boca Raton, FL., 2013.

(2) UniProtConsortium, T. UniProt: The Universal Protein Knowledgebase in 2021. *Nucleic Acids Res.* **2021**, *49* (D1), D480–D489. https://doi.org/10.1093/nar/gkaa1100.

(3) Berman, H. M.; Henrick, K.; Nakamura, H. Announcing the Worldwide Protein Data Bank. *Nat. Struct. Biol.* **2003**, *10* (12), 980.

(4) Schrödinger, L. The PyMOL Molecular Graphics System, Version~1.8. 2015.

(5) Israelachvili, J. N. *Intermolecular and Surface Forces*, Third.; Academic Press, 2011.

(6) Israelachvili, J. N.; McGuiggan, P. M. Adhesion and Short-Range Forces between Surfaces. Part I: New Apparatus for Surface Force Measurements. *J. Mater. Res.* **1990**, *5* (10), 2223–2231. https://doi.org/10.1557/JMR.1990.2223.

(7) Israelachvili, J.; Min, Y.; Akbulut, M.; Alig, A.; Carver, G.; Greene, W.; Kristiansen, K.; Meyer, E.; Pesika, N.; Rosenberg, K.; Zeng, H. Recent Advances in the Surface Forces Apparatus (SFA) Technique. *Reports Prog. Phys.* **2010**, *73* (3). https://doi.org/10.1088/0034-4885/73/3/036601.

(8) Kramer, R. Z.; Venugopal, M. G.; Bella, J.; Mayville, P.; Brodsky, B.; Berman, H. M.

Staggered Molecular Packing in Crystals of a Collagen-like Peptide with a Single Charged Pair. *J. Mol. Biol.* **2000**, *301* (5), 1191–1205. https://doi.org/10.1006/jmbi.2000.4017.

(9) Kramer, R. Z.; Bella, J.; Mayville, P.; Brodsky, B.; Berman, H. M. Sequence Dependent Conformational Variations of Collagen Triple-Helical Structure. *Nat. Struct. Biol.* **1999**, *6* (5), 454–457. https://doi.org/10.1038/8259.

(10) Hua, C.; Zhu, Y.; Xu, W.; Ye, S.; Zhang, R.; Lu, L.; Jiang, S. Characterization by High-Resolution Crystal Structure Analysis of a Triple-Helix Region of Human Collagen Type III with Potent Cell Adhesion Activity. *Biochem. Biophys. Res. Commun.* **2019**, *508* (4), 1018–1023. https://doi.org/10.1016/j.bbrc.2018.12.018.

(11) Berisio, R.; Vitagliano, L.; Mazzarella, L.; Zagari, A. Crystal Structure of the Collagen Triple Helix Model [(Pro-Pro-Gly)10]3. *Protein Sci.* **2009**, *11* (2), 262–270. https://doi.org/10.1110/ps.32602.
